# Retrospective validation study of an artificial neural network-based preoperative decision-support tool for noninvasive lymph node staging (NILS) in women with primary breast cancer (ISRCTN14341750)

**DOI:** 10.1186/s12885-024-11854-1

**Published:** 2024-01-16

**Authors:** Ida Skarping, Julia Ellbrant, Looket Dihge, Mattias Ohlsson, Linnea Huss, Pär-Ola Bendahl, Lisa Rydén

**Affiliations:** 1 Department of Clinical Sciences Lund, Division of Surgery, Lund University, Lund, Sweden; 2 Department of Clinical Physiology and Nuclear Medicine, Skåne University Hospital, Lund, Sweden; 3 Department of Surgery, Skåne University Hospital, Malmö, Sweden; 4 Department of Plastic and Reconstructive Surgery, Skåne University Hospital, Malmö, Sweden; 5 Department of Astronomy and Theoretical Physics, Division of Computational Biology and Biological Physics, Lund University, Lund, Sweden; 6 Division of Surgery, Department of Clinical Sciences Helsingborg, Lund University, Lund, Sweden; 7 Department of Surgery, Helsingborg General Hospital, Helsingborg, Sweden; 8 Division of Oncology, Department of Clinical Sciences, Lund University, Lund, Sweden; 9 Department of Surgery and Gastroenterology, Skåne University Hospital, Malmö, Sweden

**Keywords:** Breast neoplasm, Artificial neural network, Staging, Axillary lymph nodes, Validation, Decision support tool, Sentinel lymph node biopsy

## Abstract

**Background:**

Surgical sentinel lymph node biopsy (SLNB) is routinely used to reliably stage axillary lymph nodes in early breast cancer (BC). However, SLNB may be associated with postoperative arm morbidities. For most patients with BC undergoing SLNB, the findings are benign, and the procedure is currently questioned. A decision-support tool for the prediction of benign sentinel lymph nodes based on preoperatively available data has been developed using artificial neural network modelling.

**Methods:**

This was a retrospective geographical and temporal validation study of the noninvasive lymph node staging (NILS) model, based on preoperatively available data from 586 women consecutively diagnosed with primary BC at two sites. Ten preoperative clinicopathological characteristics from each patient were entered into the web-based calculator, and the probability of benign lymph nodes was predicted. The performance of the NILS model was assessed in terms of discrimination with the area under the receiver operating characteristic curve (AUC) and calibration, that is, comparison of the observed and predicted event rates of benign axillary nodal status (N0) using calibration slope and intercept. The primary endpoint was axillary nodal status (discrimination, benign [N0] vs. metastatic axillary nodal status [N+]) determined by the NILS model compared to nodal status by definitive pathology.

**Results:**

The mean age of the women in the cohort was 65 years, and most of them (93%) had luminal cancers. Approximately three-fourths of the patients had no metastases in SLNB (N0 74% and 73%, respectively). The AUC for the predicted probabilities for the whole cohort was 0.6741 (95% confidence interval: 0.6255–0.7227). More than one in four patients (*n* = 151, 26%) were identified as candidates for SLNB omission when applying the predefined cut-off for lymph node-negative status from the development cohort. The NILS model showed the best calibration in patients with a predicted high probability of healthy axilla.

**Conclusion:**

The performance of the NILS model was satisfactory. In approximately every fourth patient, SLNB could potentially be omitted. Considering the shift from postoperatively to preoperatively available predictors in this validation study, we have demonstrated the robustness of the NILS model. The clinical usability of the web interface will be evaluated before its clinical implementation.

**Trial registration:**

Registered in the ISRCTN registry with study ID ISRCTN14341750.

Date of registration 23/11/2018.

**Supplementary Information:**

The online version contains supplementary material available at 10.1186/s12885-024-11854-1.

## Introduction

To provide breast cancer (BC) patients with optimal care, axillary staging, as well as the investigation of the biological and genetic features of the tumor, are of utmost importance [1, 2]. When diagnosed, the most intrusive questions are the curability of the BC and how advanced the disease is. Surgical sentinel lymph node biopsy (SLNB) is routinely used for reliable axillary staging. Axillary imaging in early BC, most commonly ultrasound, is considered an inadequate staging modality; the inability to safely distinguish between no/low (1–2 involved axillary nodes) and high nodal burden (≥ 3 positive nodes) is particularly concerning [3]. Although considered a minor surgical procedure, SLNB is associated with considerable early and late side-effects in some patients, for example, postoperative swelling, arm lymphedema, paresthesia, and arm discomfort [4, 5]. In addition, for most BC patients undergoing SLNB, the findings are benign; hence, the procedure could have been avoided in these women [6, 7].

In the 1990s, the introduction of SLNB in routine clinical practice [8] significantly reduced axillary lymph node dissection (ALND)-associated morbidity, without compromising the long-term prognosis of these women [9]. Following the findings of axillary lymph node metastases on SLNB, patients were recommended to undergo ALND. In the pursuit of de-escalating axillary surgery, the American College of Surgeons Oncology Group (ACOSOG) Z0011 trial investigated whether ALND could be avoided in patients with clinical T1–T2 and one or two positive sentinel lymph node(s), considering adjuvant treatments and breast irradiation. This practice-changing trial showed that there was no significant difference in the locoregional recurrence rate after 10 years of follow-up [10, 11].

Currently, the most common standard axillary staging method for women with clinically node-negative (cN0) BC is SLNB, a surgical method with an established false-negative rate of 10% [12, 13]. However, the routine use of SLNB has recently been disputed. The ongoing sentinel node vs. observation after axillary ultrasound (SOUND) trial is comparing SNLB vs. non surgical staging of the axilla in women with cN0 BC and tumors < 20 mm [14]. In addition, in an ongoing Dutch multicenter trial, the Borstkanker Onderzoek Groep (BOOG) 2013–08 trial, the safety of SLNB omission in women with cN0 T1–2 invasive BC undergoing breast-conserving surgery was investigated [15]. In addition with the results from the ongoing Intergroup Sentinel Mamma (INSEMA) trial [7], hopefully, there will soon be enough data available to support SLNB omission in patients fulfilling the inclusion criteria for these trials without reducing oncological safety. Recently, patient-reported outcomes in the INSEMA trial have been published, showing less arm morbidity in the non-SLNB group than in the SLNB group [16]. Further de-escalation is supported by the updated American Society of Clinical Oncology (ASCO) guidelines presented in 2021, stating that SLNB could be omitted altogether in women ≥ 70 years with cT1N0, hormone receptor-positive, human epidermal growth factor receptor 2 (HER2)-negative BC, conditionally treated with adjuvant hormonal therapy [2]. Concurrently, the indication for neoadjuvant chemotherapy has broadened, including patients with triple-negative BC and HER2-positive BC; in patients with pathological complete response, the omission of SLNB after neoadjuvant chemotherapy is now evaluated (EUBREAST-01 ClinicalTrials.gov Identifier: NCT04101851) [1, 17].

We developed an artificial neural network (ANN) model for noninvasive lymph node staging (NILS) for early cN0 BC. In 2019, the original ANN model utilized 15 clinical and postoperative pathological characteristics for predicting nodal status [18] and showed an estimated potential to reduce the fraction of SLNB by 18–27% in newly diagnosed BC patients. Implementing the NILS prediction model is cost-effective, according to health-economy modelling; the NILS prediction model has been shown to be associated with cost reductions and likely overall health gains [19]. To provide a clinically useful risk assessment of nodal involvement, 10 preoperatively available variables have been used in the current updated NILS model for which a web interface has been developed [20]. Herein, we present the results of the NILS validation study using exclusively preoperative data for risk assessment. The study protocol was published before the finalization of data collection, including sample size estimation [21].

The present study aimed to validate the NILS prediction model using one temporal and one geographical retrospective cohort. The important development from the original ANN model, including preoperative and postoperative input characteristics, to the current NILS model, strictly using preoperative data only, constitutes domain validation.

## Methods

Women, ≥18 years of age, with cN0 (clinically and by ultrasound) invasive BC diagnosed through core needle biopsy (CNB) scheduled for primary surgery and accepting participation (i.e., not opting out) were included. Surgical axillary staging with SLNB was a prerequisite for inclusion in this study. Patients previously undergoing ipsilateral breast/axillary surgery, scheduled for neoadjuvant chemotherapy, or undergoing upfront axillary nodal dissection were not eligible for study inclusion.

In this retrospective, observational cohort study, retrieved patient and tumor characteristics were prospectively entered into the web calculator. No interventions or additional examinations were performed, and no results were reported to the patients or physicians. The patients were treated according to clinical routine and oncological treatment in accordance with decisions made at the multidisciplinary conference, and the resected breast and axillary lymph nodes were pathologically examined. The STrengthening the Reporting of OBservational studies in Epidemiology (STROBE) checklist [22] was used for study and manuscript preparation.

### The NILS prediction model

The study population for the development of the ANN model was based on a prospectively maintained pathological database on consecutive breast cancer patients with clinically node negative status scheduled for primary surgery at Skåne University Hospital, Lund, Sweden. The original ANN model presented in 2019 was based on a combination of preoperatively and postoperatively available data [18]. The characteristics of the development cohort is thoroughly described in the provided Supplementary File 1.

In short, ANN is a machine learning method that has the ability to explore nonlinear associations in a dataset. The ANN within the NILS prediction model contains ensembles of multilayer perceptrons (MLP). Each MLP contains three layers: 1) the input layer (clinicopathological variables), 2) the hidden layer, and 3) the output layer. To learn the association to nodal status (output), the MLPs were trained by a standard back-propagation technique. Four-fold cross-validation, repeated five times, was used as the internal model validation strategy to obtain optimal model parameters.

**Fig. 1:**
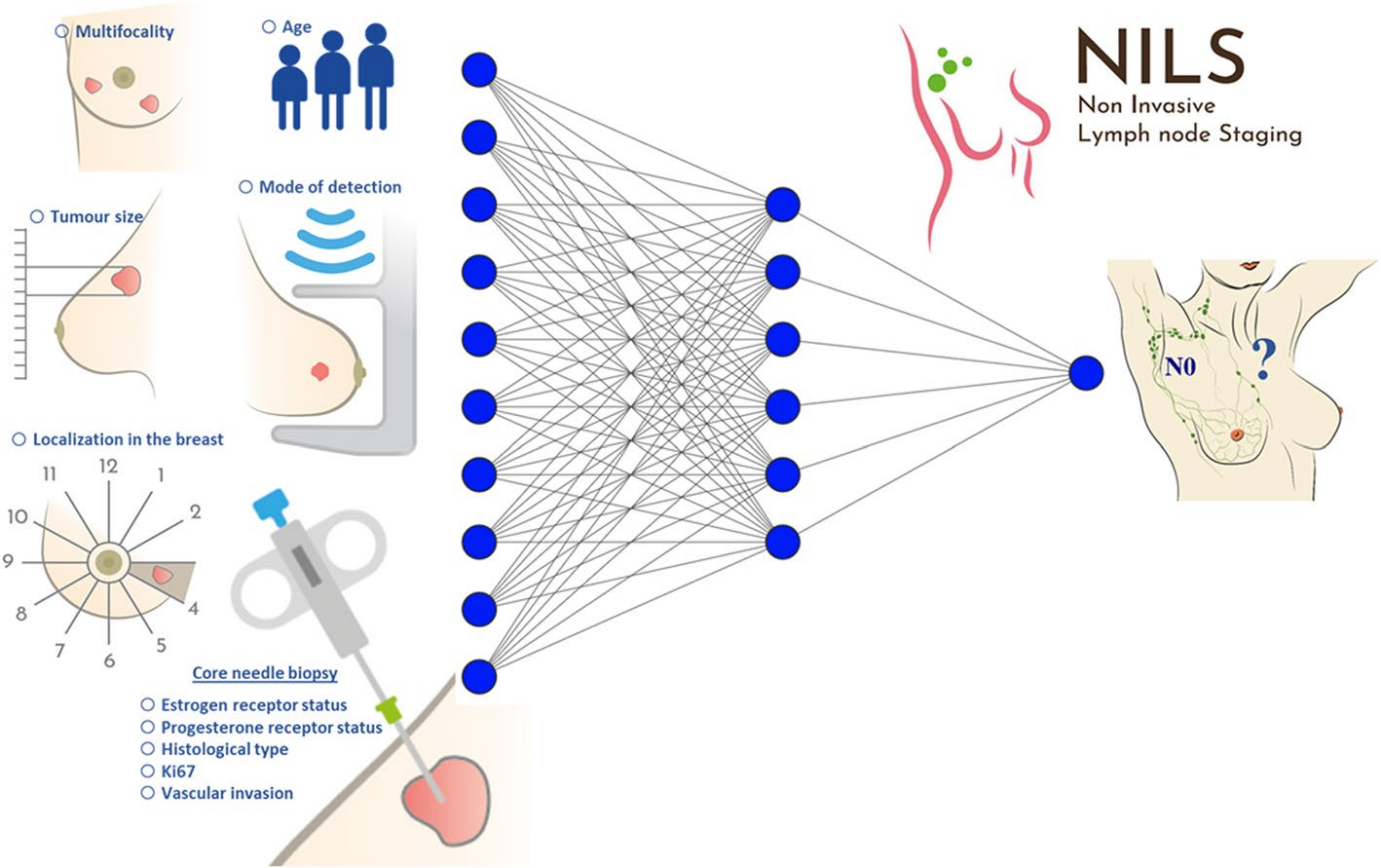
Schematic figure of included variables

In this study, the NILS model for the prediction of nodal status in cN0 BC patients was based on 10 preoperatively available features: patient age at diagnosis and eight features that are easily and reliably accessible from preoperative imaging and CNBs: tumor size, multifocality, estrogen receptor (ER) status, progesterone receptor (PR) status, histological type, mode of detection, tumor localization in the breast, and Ki-67 positivity. Vascular invasion, the tenth feature of the NILS model, was difficult to determine preoperatively (Fig. 1). Therefore, a separate ANN model was developed to impute this feature, using the other nine features of the NILS model as predictors. The model reflects routine diagnostic BC workup and was trained to handle missing histopathological input variables. NILS handles combinations of unknown LVI status and missing values for ER/PR status and the proliferation index Ki67; however, the six remaining variables (age, mode of detection, tumor localization in the breast, tumor size, multifocality, and histological type) were mandatory. A user-friendly web implementation of the NILS model was tested in this study. Data from patient records were extracted and used as inputs to predict the axillary nodal status. For each patient, the output from the calculator displays the estimated probability of healthy lymph nodes as well as the malignant or benign categorization of the nodes [20]. Although strictly preoperative variables were used for the estimation of the probability of healthy lymph nodes using the NILS web interface, supplementary postoperative variables (10 input variables) were acquired, enabling a batch-mode validation of the original model (thus not using the web interface).

### Cohort

**Fig. 2:**
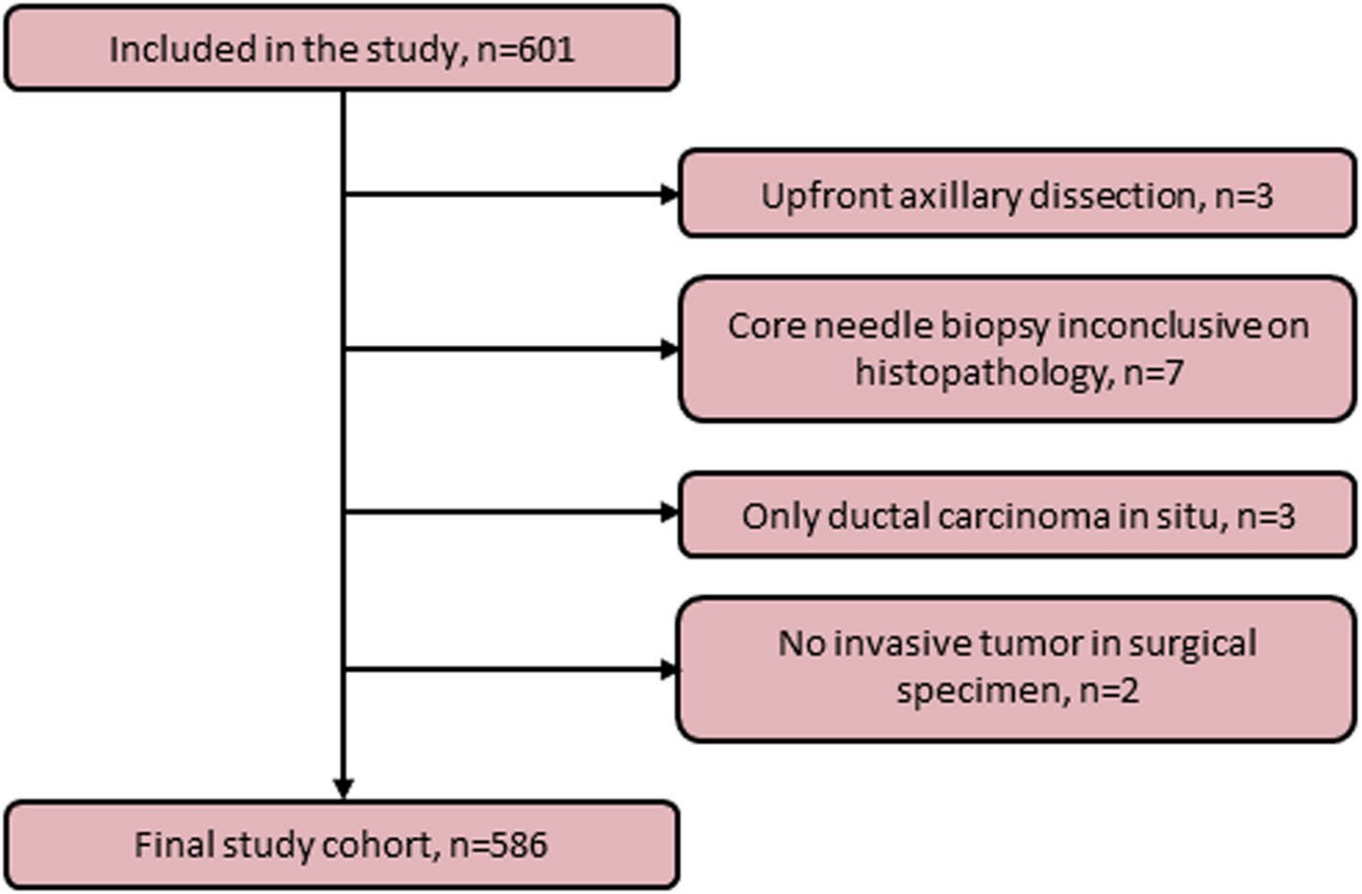
Flow chart

A total of 601 patients who underwent surgery for primary BC at two sites were included: 401 at site 1 (Malmö, Skane University Hospital, Sweden in 2020), constituting a temporal validation cohort, and 200 at site 2 (Helsingborg Regional Hospital, Sweden, between 2019 and 2020), constituting a geographical validation cohort. Patients were identified through the national registry of cancer diagnoses, treatments, and outcomes (the Swedish National Quality Registry for Breast Cancer [23]). Digital medical charts were reviewed by three experienced research nurses and two physicians (*n* = 5 in total). The inclusion and exclusion criteria strictly followed the study protocol [21]. Fifteen patients were excluded because they did not meet the inclusion criteria (Fig. 2).

cN0 was defined as no axillary nodal involvement on clinical or radiological examination. pN0 was defined as no invasive cell clusters of > 0.2 mm at the largest diameter in the lymph node (i.e., the presence of isolated tumor cells or cell clusters that are ≤ 0.2 mm at the largest diameter are considered pN0).

### Data management

In this validation study, we used the Research Electronic Data Capture (REDCap) module using audit trail for data management [24]. Thorough data monitoring was performed by an independent researcher not involved in data entry, according to a predefined flow chart and quality control plan (Supplementary Material S1 [21]). In addition, the study statistician (POB) checked the entered data to identify inconsistencies not captured by the data entry rules, as defined in the REDCap. The low number of missing data points was due to meticulous data management.

### Statistical analysis

Prior to study initiation, sample size calculation was performed as described in the study protocol [21]. Descriptive statistics were presented for the whole cohort and split according to the study site. Chi-squared tests and t-tests were used, where appropriate, to test for homogeneity across sites. The performance of the NILS model was assessed in terms of discrimination with the area under the receiver operating characteristic (ROC) curve (AUC) and calibration, that is, comparison of the observed and predicted event rates of axillary disease using Hosmer–Lemeshow graphics summarized by calibration slope and intercept [25]. Perfect calibration corresponds to a slope of 1 and an intercept of 0. Locally weighted scatterplot smoothing (LOWESS) was used to capture the calibration performance for high probabilities of N0, the range of interest for a de-escalation strategy.

The primary endpoint was axillary nodal status (discrimination, N0 vs. N+) determined by the NILS model (test result) in comparison with nodal status by definitive pathological diagnosis after SLNB. As specified in the study plan, subgroup analyses were performed according to: 1) study site; 2) complete data on all CNB features in NILS (vascular invasion was excluded because of the routine unavailability of this variable preoperatively) or records with imputed values; 3) mastectomy or breast-conserving surgery; and 4) separately for ER-positive/HER2-negative, T1-2, and age ≥ 70 (subgroup specified by Choosing Wisely and adopted by ASCO guidelines on axillary staging [2]). Test characteristics in terms of sensitivity, specificity, false positive rate (FPR), and false negative rate (FNR) were computed. NILS indicating nodal disease (N+) was defined as ”positive” [1] in coherence with N+ as assessed by pathology (Supplementary File 2). The FNR in the NILS model was calculated as the number of false N0 cases predicted by NILS divided by the number of cases with pathology-verified axillary nodal metastasis. Finally, clinical utility was evaluated as the proportion of patients for whom SLNB could have been avoided and by decision curve analysis as a net benefit [26] for NILS compared to SLNB for all patients in the target population.

In addition, the original ANN model, which included both preoperatively and postoperatively available variables, was validated in batch-mode, thus bypassing the web interface (preoperative variables: patient age at diagnosis, mode of detection, and tumor localization in the breast; postoperative variables: tumor size, multifocality, ER status, PR status, histological type, Ki67 value, and vascular invasion).

All analyses were performed using the Statistical Package for the Social Sciences Statistics for Windows, version 26 (IBM Corp., Armonk, NY, USA) and StataCorp. 2021. Stata Statistical Software: Release 17. Decision curve analysis was performed in Stata using dca.ado and custom-made software written in C (gcc version 7.5.0) and Perl (version 5.26.1).

### Ethical approval

All procedures performed in this study involving human participants were in accordance with the ethical standards of the institutional or national research committee and with the 1964 Declaration of Helsinki and its later amendments or comparable ethical standards. This study was approved by the Swedish Ethical Review Authority (Ethical Review Board, Stockholm Department 3 Medicine, committee reference number: 2021–00174). Previously treated patients received study information through advertisements in the local press and were allowed to opt out by using the provided contact information. The ethics committee (Ethical Review Board, Stockholm Department 3 Medicine, committee reference number: 2021–00174) waived the requirement for informed consent and consent for publication. As per Swedish law, all patients receiving treatment for BC at hospitals are to provide consent for registration in a national registry of cancer. The local department for personal data admission at the hospital (KVB Samråd, Region Skåne, Sweden) granted researchers access, with digital logging, to patients’ digital medical charts. Only users authorized by the principal investigator had access to the NILS web calculator.

## Results

### Descriptive statistics

The mean age of the patients in the cohort at diagnosis was 65 years; thus, the majority of the women were postmenopausal (85%) (Table 1). Most patients had unifocal (91% on mammography), ER-positive (93%), PR-positive (82%), and HER2-negative (95%) tumors (according to preoperative CNB). The mean tumor size on mammography was 19 mm, with slightly larger tumors at site 2 than at site 1 (mean 17 mm vs. 22 mm, *p*-value: < 0.001). Data on ultrasound variables are shown in Supplementary File 3. Approximately three-fourths of the patients had no metastases in SLNB (N0 74%). Of the patients with metastases on SLNB (N+), the majority had macrometastases (Table 2). The SLNB reduction rate was 26% (Table 3). A comparison between the current validation and original cohorts, in which the NILS model was first developed, is presented in Supplementary File 1.

**Table 1:** Patient and tumor characteristics at baseline, total and by study site

		Total		Site 1		Site 2		
		Count	%	Count	%	Count	%	*p*-value*
Number of patients (NB! Row percent)		586		395	67.4%	191	32.6%	
Age at diagnosis, years	Mean (range)	65 (29—91)		64 (30—91)		65 (29—90)		0.451
BMI	Mean (range)	26.8 (16.7—48.9)		27.2 (16.7—48.9)		26.2 (18.8—42.9)		0.020
	Missing	50		49		1		
Postmenopausal	No	85	15.4%	55	14.9%	30	16.5%	0.620
	Yes	467	84.6%	315	85.1%	152	83.5%	
	Missing	34		25		9		
Screening detected	No	240	41.0%	155	39.2%	85	44.5%	0.225
	Yes	346	59.0%	240	60.8%	106	55.5%	
Bilateral cancer	No	553	94.4%	379	95.9%	174	91.1%	0.017
	Yes	33	5.6%	16	4.1%	17	8.9%	
Multifocal cancer (mammography)	No	531	91.1%	359	91.1%	172	91.0%	0.965
	Yes	52	8.9%	35	8.9%	17	9.0%	
	Missing^a^	3		1		2		
Largest tumor (long axis, mm, mammography)	Mean (range)	19 (3–110)		17 (3–110)		22 (5–90)		< 0.001
	Missing^a^	55		30		25		
Centrally positioned tumor (mammography, sub areolar or within 2 cm of the mammilla)	No	383	87.4%	251	88.1%	132	86.3%	0.589
	Yes	55	12.6%	34	11.9%	21	13.7%	
	Missing^a^	148		110		38		
ER status CNB	Negative (< 1%)	24	7.3%	20	9.4%	4	3.4%	0.048
	Positive (≥ 1%)	305	92.7%	193	90.6%	112	96.6%	
	Missing	257		182		75		
PR status CNB	Negative (< 1%)	57	18.0%	40	19.3%	17	15.6%	0.413
	Positive (≥ 1%)	259	82.0%	167	80.7%	92	84.4%	
	Missing	270		188		82		
HER2 status CNB	Negative	296	95.2%	194	94.6%	102	96.2%	0.534
	Positive	15	4.8%	11	5.4%	4	3.8%	
	Missing	275		190		85		
Proliferation index Ki67 (%) CNB	Mean (range)	26.6 (1.0—95.0)		28.4 (3.0—95.0)		23.1 (1.0—83.0)		0.016
	Missing	274		189		85		
Complete cases ER/PR/Ki67 CNB	No	274	46.8%	189	47.8%	85	44.5%	0.453
	Yes	312	53.2%	206	52.2%	106	55.5%	
Vascular invasion CNB (only reported when present)	Yes	4		4		0		-
	Missing	582		391		191		
Histopathological type CNB	NST (No specific type, ductal)	445	75.9%	301	76.2%	144	75.4%	0.705
	Lobular	105	17.9%	72	18.2%	33	17.3%	
	Other	36	6.2%	22	5.6%	14	7.3%	

^*^For categorical variables, Chi-square test was used and for continuous variables, t-test was used

^a^When missing data on mammography, features from ultrasound was entered into the NILS web interface

*Abbreviations BMI* Body mass index, *CNB* Core needle biopsy, *ER* Estrogen receptor, *PR* Progesterone receptor, *HER2* Human epidermal growth factor receptor 2

**Table 2:** Characteristics of sentinel lymph node(s), total and by study site

		Total		Site 1		Site 2		
		Count	%	Count	%	Count	%	*p* value*
Number of extracted SLN(s)	1	158	27.0%	90	22.8%	68	35.6%	< 0.001
	2	198	33.8%	124	31.4%	74	38.7%	
	3 +	230	39.2%	181	45.8%	49	25.7%	
Metastases in SLN	No	432	73.7%	293	74.2%	139	72.8%	0.718
	Yes	154	26.3%	102	25.8%	52	27.2%	
Number of metastases in SLN(s)	1	108	70.1%	68	66.7%	40	76.9%	0.313
	2	33	21.4%	25	24.5%	8	15.4%	
	3	10	6.5%	6	5.9%	4	7.7%	
	4	3	1.9%	3	2.9%	0	0.0%	
Size largest SLN-metastasis (mm), mean (range)		5.2 (0.2—38.0)		5.1 (0.2—38.0)		5.3 (0.3—26.0)		0.857
SLN metastases, categorized	No metastases	432	73.7%	293	74.2%	139	72.8%	0.928
	Micro: > 0.2 mm, ≤ 2 mm	64	10.9%	42	10.6%	22	11.5%	
	Macro: > 2 mm	90	15.4%	60	15.2%	30	15.7%	

^*^For categorical variables, Chi-squared test was used and for continuous variables, t-test was used

*Abbreviations*: *SLN* Sentinel lymph node

**Table 3:** Comparison between performance measures of the noninvasive lymph node staging (NILS) model, including potential sentinel lymph node biopsy (SLNB) reduction rates between the original ANN model and current model

Model	TP	TN	FP	FN	Sensitivity	Specificity	FNR^a^	SLNB reduction rate^b^
ANN Prototype (2019)	258	190	324	28	91%	37%	10%	27%
NILS (1.0)	256	177	339	28	90%	34%	10%	26%
NILS (version 0.1.0) Validation	136	133	299	18	88%	31%	12%	26%

^a^Equivalent to the maximum FNR of 10% reflecting accepted FNR of the SLNB procedure

^b^The SLNB reduction rate was calculated as follows = (TN + FN)/(TN + FN + TP + FP)

*Abbreviations*: *TP* True positive, *TN* True negative, *FP* False positive, *FN* False negative, *FNR* False negative rate, *SLNB* Sentinel lymph node biopsy

### Discrimination, calibration, and net benefit

**Fig. 3:**
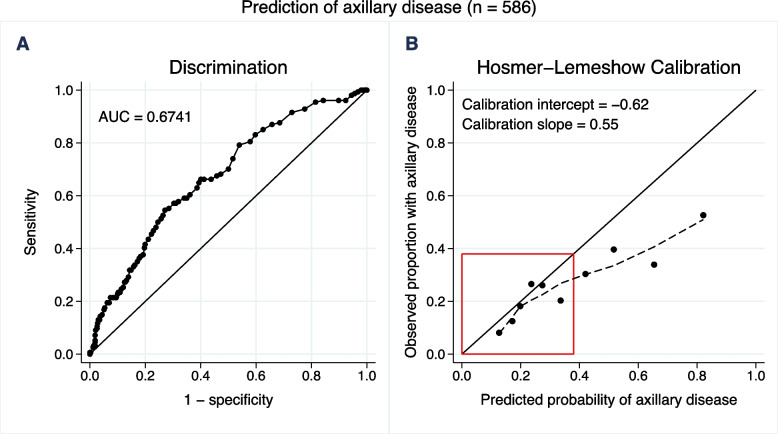
The entire cohort. A) Area under the receiver operating characteristics curve (AUC) visualizing discriminatory performance of noninvasive lymph node staging (NILS) model for the estimation of axillary disease (N+). B) The Hosmer–Lemeshow calibration plot of observed proportion N+ versus mean predicted probability of N+ for each decile of the predictions. Locally weighted scatterplot smoothing (LOWESS), the dotted line, was used to capture the calibration performance for low probabilities of N+ , i.e. within the red box

**Fig. 4:**
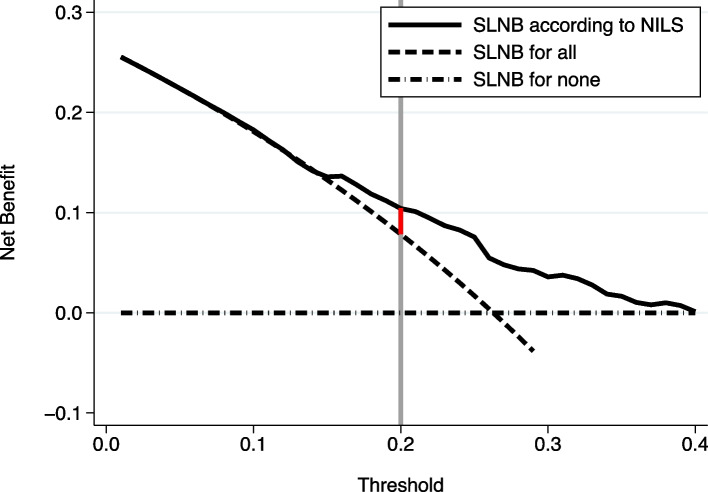
Decision Curve Analysis: Net benefit for noninvasive lymph node staging (NILS) model and the strategies sentinel lymph node biopsy (SLNB) for all and for none. The vertical grey line shows the cut-off 20% for N+ which in decision curve analysis terminology corresponds to accepting four false positive tests for each true positive test. The vertical red line represents the expected gain in net benefit if SLNB for all is replaced by the NILS model

The discriminatory ability of the NILS model was assessed with ROC analysis: the AUC for the entire cohort was 0.6741 (95% confidence interval [CI]: 0.6255–0.7227) (Fig. 3.) In the subset of women aged ≥ 70 years with ER-positive/HER2-negative tumors (*n* = 113), the AUC was 0.6006 (95% CI: 0.4888–0.7124) (Supplementary Fig. 1). The NILS model showed the best calibration in patients with a high estimated probability of healthy axilla (lower left corner of Fig. 3 B). In the entire cohort, using the predefined cut-off, the sensitivity of the NILS model was 88% (136/154), corresponding to a FNR of 12% (18/154). The specificity was 31% (133/432), and 151 of the 586 patients (26%) could potentially have been spared SLNB if the NILS model had been used. The net benefit [26] of the NILS model compared to the SLNB strategies for all and none is shown in Fig. 4. The vertical line at the threshold of 20% of being N+ corresponds to accepting four false-positive tests for each true-positive test. The difference in net benefit at this cut-off between using the NILS model and the strategy SLNB to all was 0.026, which should be interpreted as 2.6 more true positives in favor of the NILS model for every 100 patients in the target population. Furthermore, it is clear from the Fig. 4 that, as measured by net benefit, NILS is superior to SLNB for all patients in a wide range of cut-offs, approximately 20%. The cut-off value of 20% highlighted in Fig. 4 is a compromise between the predefined separate thresholds for patients with and without imputed biomarker data, which are slightly above and below 20%, respectively.

**Fig. 5:**
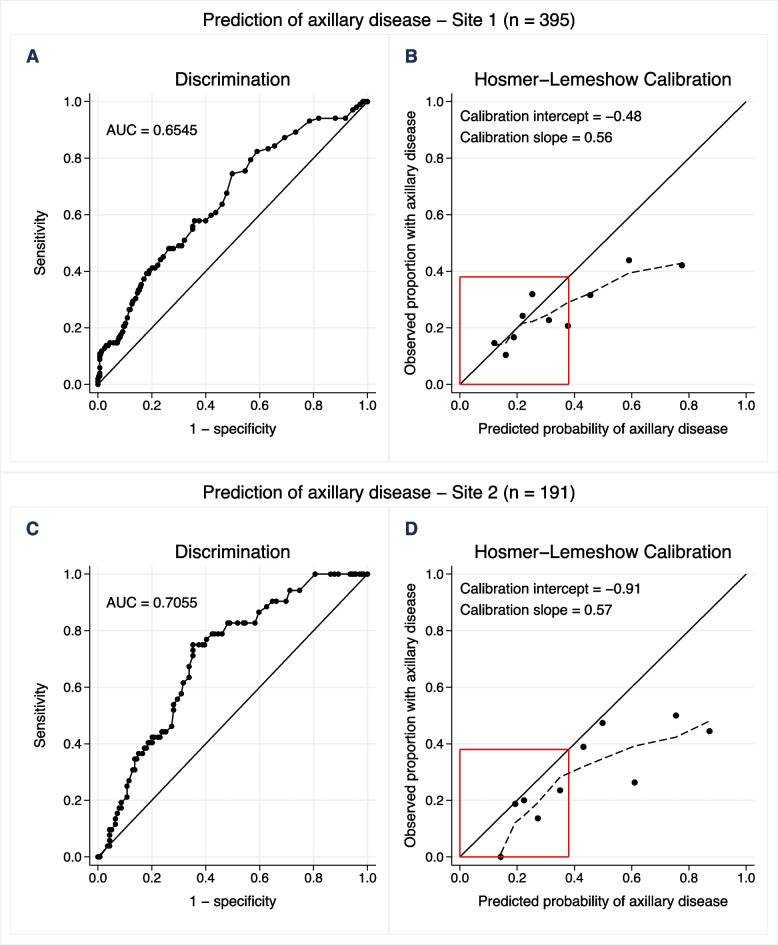
Geographical validation – results per site. A/C) Area under the receiver operating characteristics curve (AUC) visualizing discriminatory performance of noninvasive lymph node staging (NILS) model for the estimation of axillary disease (N+). B/D) Hosmer–Lemeshow calibration plot of observed proportion N+ versus mean predicted probability of N+ for each decile of the predictions. Locally weighted scatterplot smoothing (LOWESS), the dotted line, was used to capture the calibration performance for low probabilities of N+ , i.e. within the red box

**Fig. 6:**
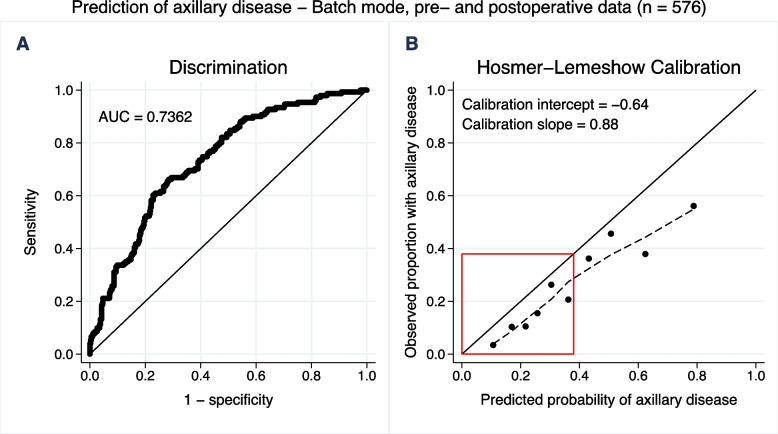
The entire cohort, batch-mode results using both preoperative and postoperative data. A) Area under the receiver operating characteristics curve (AUC) visualizing discriminatory performance of noninvasive lymph node staging (NILS) model for the estimation of axillary disease (N+). B) Hosmer–Lemeshow calibration plot of observed proportion N+ versus mean predicted probability of N+ for each decile of the predictions. Locally weighted scatterplot smoothing (LOWESS), the dotted line, was used to capture the calibration performance for low probabilities of N+ , i.e. within the red box

The discriminatory performance was similar for sites 1 and 2 (Fig. 5). Likewise, a similar discriminatory performance was noted in patients with complete data on biomarkers (except for vascular invasion) and in those with imputed biomarkers (Supplementary Fig. 2). The batch-mode validation, utilizing a combination of preoperative and postoperative variables, showed an AUC of 0.7362 (95% CI: 0.6917–0.7807) for discrimination of N0 vs N+ (Fig. 6). The discrimination in terms of AUC for nodal prediction in the breast surgery-based prespecified subgroup analyses based is provided in Supplementary Fig. 3.

## Discussion

In this temporal and geographical validation study, considering the clinically important shift from a combination of preoperative and postoperative variables to strictly preoperative variables, the NILS decision support tool showed a satisfactory performance. The validation study demonstrated the potential to abstain from axillary surgery in 26% of the patients with cN0 BC using NILS, corresponding to the identified proportion of 27% in the developmental cohort. Altogether, the case-by-case preoperative evaluation provided by the NILS decision support tool holds clinical utility in the near future.

When developing a prediction model, the overall aim is that the model should be valid in the target population outside the developmental area and in patients with slightly modified characteristics [27–30]. Therefore, in this study, we performed a thorough temporal and geographical validation study and used preoperatively available variables instead of a combination of preoperatively and postoperatively available variables.

We present the results of this validation study of a preoperative ANN model for noninvasive nodal staging in early breast cancer [20]. The study protocol was previously published, along with a rigorous data management plan, thus increasing the validity of the current study results. In the present validation study, the NILS model performed best in low-risk BC patients presenting with the highest estimated probability of healthy lymph nodes (N0), and the calibration for these patients was considerably better than the overall calibration (indicated by the red boxes in the calibration graphs). In these patients, the NILS decision support tool has the potential to reduce the number of SLNBs performed, with an SLNB reduction rate of 26%. To the best of our knowledge, the NILS model is the first predictive model for nodal status in early BC with a user-friendly web interface. This validation study is an important step towards making the NILS model clinically available.

The preoperative (core needle biopsy) distribution of immunohistochemical receptors and HER2 status displayed a high proportion of ER-positive/HER2-negative tumors, which is expected given the inclusion/exclusion criteria: patients with triple-negative and HER2-positive tumors are primarily recommended to receive neoadjuvant chemotherapy [1] and were thus not eligible for this study. The majority of patients included in this study, therefore, presented with luminal BC eligible for upfront surgical treatment; in addition, these patients make up the population intended for the NILS clinical decision support tool.

Different summary measures have been suggested for evaluating the performance of a diagnostic test. Depending on the purpose of the test, each of these measures is more or less appropriate. In addition, both calibration and discrimination can be of different importance in separate patient populations. In the present validation study, the most important population was women presenting with low-risk tumors, in whom SLNB can be avoided. In the present study, we showed the best calibration in patients with the highest probability of having a healthy axilla, namely, the patients we aimed to identify with the NILS model. Furthermore, we purposely developed a conservative model to keep the FNR (the NILS model recommends abstaining from SLNB in N+ patients) at an acceptable and oncologically safe rate. We consider the identified FNR of the NILS model of 12% in this validation study to be of the same magnitude as that of the reference SLNB (approximately 10% [12, 13]), and thus acceptable.

As for many clinical prediction models in oncology, the NILS prediction model aims to achieve a high sensitivity (90%) to avoid harms of missed malignant nodal metastasis. As sensitivity and specificity are inversely proportional, a high sensitivity comes at the cost of a lower specificity. In a clinical context, if the NILS prediction model is used as a clinical decision-support tool, patients with a healthy axilla will undergo an (unnecessary) SLNB in order to maintain a low false negative rate. In model development, the threshold at which the level of sensitivity/specificity is set, can easily be modified using a different cut-off to balance benefits and harms. However, in the NILS prediction model, all decisions on thresholds were based on established conservative clinical cut-offs; as such, we presented a model with an oncologically safe prediction of nodal status with demonstrated cost effectiveness.

Geographical, temporal, and domain validation of a predictive model is warranted before clinical implementation. This step is often overlooked; however, as an example of its importance, when the Memorial Sloan-Kettering Cancer Center nomogram for prediction of nodal status was externally validated, the AUC dropped from 0.75 to AUC 0.67 (95% CI 0.63–0.72) [31, 32].

The AUC is a global measure focused on the predictive performance of a given model over the full range of the risk spectrum. However, the consequences of the decisions are not further considered. Decision curve analysis estimates the “net benefit” (the clinical utility) of the NILS model in comparison to the default strategy, that is, SLNB for all patients [26]. At the predefined “exchange rate” 1:4 between true and false positives, NILS was shown to have a higher net benefit than SLNB for all patients.

The attained AUC in the NILS prediction model is comparable to previously reported models including only routine clinicopathological data for nodal status prediction (AUC 0.67–0.79) [31–33]; however, there is a lack of reports on possible SLNB reduction rates and estimates on cost effectiveness linked to model performance.

The patients included at site 2 (the geographical validation cohort) had larger tumors than those treated at site 1 (the temporal validation cohort). This is explained by the fact that at site 2, fine needle aspiration is routinely used for cancer diagnosis of breast tumors in low-risk patients, including those with a suspected malignancy < 2 cm in size. Since data on histopathological subtypes from CNB are mandatory in the NILS model, these low-risk patients were excluded, shifting the population at site 2 to a cohort with larger tumors. However, the proportion of N+ was similar between the two sites. Interestingly, the NILS model had better discrimination at site 2; however, the calibration was better at site 1. Whether this is a consequence of the fewer low-risk patients at site 2 needs further elaboration.

As this is a validation study, it is important to consider the original cohort [18] and method. First, in the original study, 800 patients diagnosed between 2009 and 2012 were included, whereas the patients in the current cohort were diagnosed between 2019 and 2020. Fortunately, in Sweden and internationally, the fraction of women presenting with N+ BC at diagnosis is continuously decreasing [34, 35]. In addition, the criteria for recommending neoadjuvant chemotherapy have broadened. As a result, patients undergoing primary surgery in recent years present with a less advanced BC stage, which explains some differences between the original and validation cohorts. The present study indicated that the proportion of patients presenting with N+ disease decreased from 36 to 26%. Furthermore, the original study used a combination of preoperatively and postoperatively available data, whereas the current study strictly used preoperatively available data to mimic the clinical preoperative situation in which the NILS model was applicable. Despite these differences, the NILS model showed satisfactory performance in this validation study, indicating the robustness of the model. Moreover, the batch-mode run validation using the same combination of preoperative and postoperative variables as in the original cohort showed, as expected, better discrimination in accordance with published data.

As the NILS prediction model is not intended to guide oncological treatment (with changing indications/strategies over time), using clinicopathological variables to predict nodal status, the only concern with the cohort is the changing prevalence of pN0 over time. The NILS prediction model and its web interface could easily be adjusted to different pN0 prevalence in different populations and over time, thus vastly increasing generalizability over time and place. Another way to circumvent the changing N+ BC prevalence over time is to evaluate the utility of NILS in terms of the prevalence independent measures of positive and negative likelihood ratios. Interestingly, the conservative nature of the NILS decision-support tool reflects the greatest clinical value for women with a low estimated risk of nodal metastases.

### Study strengths and limitations

Our model performed equally well when biomarkers and vascular invasion were imputed as when these data were available. The AUC was marginally higher when data were imputed; however, the calibration slope deviated more for the optimal value of 1.0. This shows the robustness of the NILS model and broadens the area of application to sites in which biomarkers are not routinely analyzed. In addition, a significant advantage was the previously published study protocol, which includes a predefined data quality and statistical plan.

The NILS prediction model is based on an ANN algorithm. However, there are other machine-learning models in addition to logistic regression models for the same purpose. In the original publication, the ANN model performed slightly better than logistic regression upon internal validation with cross-validation, and thus selected for further application and development. Variation in the proportions of node positive breast cancer in different target population may impact the performance of the NILS prediction model.

It is interesting to briefly consider the variable “screening-detected.” In Sweden, women aged 40–74 years are offered mammographic screening; thus, only women in this age group have the prospect of having screening-detected BC. However, the NILS model was developed independently of these age conditions. In addition, poorer discrimination of NILS in the population of women aged ≥ 70 years with ER-positive/HER2-negative tumors was a limitation of the present study. In future work with the NILS decision-support tool, it is possible to adjust the screening/age discrepancy and develop a model specific to the target population of women aged ≥ 70 years with ER-positive/HER2-negative tumors to better cohere with plausible future clinical settings.

### Future perspective

We performed a thorough retrospective validation of the NILS model. Concurrently, we are optimizing the web interface to improve its usability before clinical implementation.

To provide patient-centered care on the management of axilla in early breast cancer, the ASCO guideline states that patients should be evaluated on a case-by-case basis. We present a user-friendly decision-support tool for personalized pre-operative prediction of nodal status, with the potential to identify one-quarter of patients as eligible for abstaining SLNB. The adoption of prevailing guidelines can be enhanced by providing clinicians and patients with such tools. Furthermore, by conducting a health–economic analysis, the implementation of the NILS prediction model was shown to be cost-effective and associated with health gain [19].

## Conclusion

In this retrospective validation study of an ANN model, we presented the results of the NILS decision-support tool. In the temporal and geographical validation study presented here, the performance of the algorithm was satisfactory. Considering the shift from postoperatively to preoperatively available predictors in this temporal, geographical, and domain validation, we showed the robustness of the NILS model. Furthermore, we demonstrated the possibility of avoiding axillary surgery in 26% of the patients using the NILS decision-support tool. The clinical usability of the web interface will be evaluated before clinical implementation of this decision-support tool for the prediction of benign SLN(s).

### Supplementary Information

**Additional file 1. Supplementary File 1 **

**Additional file 2. Supplementary File 2**

**Additional file 3. Supplementary File 3**

**Additional file 4. Supplementary Figure 1**

**Additional file 5. Supplementary Figure 2**

**Additional file 6. Supplementary Figure 3**

## Data Availability

The raw datasets are available from the corresponding author upon reasonable request owing to privacy or ethical restrictions.

## Abbreviations

ALN: Axillary lymph node
ALND: Axillary lymph node dissection
ANN: Artificial neural network
AUC: Area under the receiver operating characteristic curve
BC: Breast cancer
CNB: Core needle biopsy
ER: Estrogen receptor
HER2: Human epidermal growth factor receptor 2
HR: Hormone receptor
LOWESS: Locally weighted scatterplot smoothing
MLP: Multilayer perceptrons
NILS: Noninvasive lymph node staging
PR: Progesterone receptor
ROC: Receiver operating characteristic
SLN: Sentinel lymph node
SLNB: Sentinel lymph node biopsy
