# Supplementary material for: Retrospective validation study of an artificial neural network-based preoperative decision-support tool for noninvasive lymph node staging (NILS) in women with primary breast cancer (ISRCTN14341750)
Source: BMC Cancer. 2024 Jan 16;24:86. doi: 10.1186/s12885-024-11854-1 (PMC10790472; doi:10.1186/s12885-024-11854-1)
Supplement: Supplementary file 1 — Additional file 1. Supplementary File 1 [file 12885_2024_11854_MOESM1_ESM.docx]

Supplementary File 1. Comparison of patient and tumor characteristics between the current cohort and the cohort in which the original NILS model was developed.

|  | | **Study cohort** | | **Dihge et al, BMC Cancer 2019** | |
| --- | --- | --- | --- | --- | --- |
|  | | Count | % | Count | % |
| N status by pathology | Negative | 432 | 74% | 514 | 64% |
|  | Positive | 154 | 26% | 286 | 36% |
| Age at diagnosis, years, median (range) | | 68 (29–91) |  | 64 (24–92) |  |
| Screening detected | No | 240 | 41% | 343 | 43% |
|  | Yes | 346 | 59% | 457 | 57% |
| Laterality | Right | 283 | 48% | 418 | 52% |
|  | Left | 303 | 52% | 382 | 48% |
| Centrally positioned tumor (mammography, sub areolar or within 2 cm of the mammilla) | No | 509 | 87% | 778 | 97% |
|  | Yes | 77 | 13% | 22 | 3% |
| Tumor size, preoperative mammography, mm, median (range) | | 20 (3.0–110) |  |  |  |
| Tumor size PAD, mm, median (range) |  | 18 (1.0–140) |  | 15 (0.5–90) |  |
|  | Missing | 0 |  | 1 |  |
| Multifocal cancer, preoperative mammography | No | 534 | 91% |  |  |
|  | Yes | 52 | 9% |  |  |
| Multifocal cancer, postoperative PAD | No | 429 | 74% | 610 | 77% |
|  | Yes | 149 | 26% | 179 | 23% |
|  | Missing | 8 |  | 11 |  |
| Vascular invasion CNB (only reported when present, not when absent) | Yes | 4 | 100% |  |  |
|  | Missing | 582 |  |  |  |
| Vascular invasion postoperative PAD | No | 470 | 82% | 545 | 85% |
|  | Yes | 103 | 18% | 94 | 15% |
|  | Missing | 13 |  | 161 |  |
| Histopathological type CNB | NST (No specific type, ductal) | 445 | 76% |  |  |
|  | Lobular | 105 | 18% |  |  |
|  | Other | 36 | 6% |  |  |
| Histopathological type PAD | NST (No specific type, ductal) | 428 | 73% | 640 | 80% |
|  | Lobular | 114 | 19% | 101 | 13% |
|  | Other | 44 | 8% | 59 | 7% |
| ER status CNB | Negative (<1%) | 24 | 7% |  |  |
|  | Positive (≥1%) | 305 | 93% |  |  |
|  | Missing | 257 |  |  |  |
| ER status PAD | Negative (<1%) | 36 | 6% | 69 | 9% |
|  | Positive (≥1%) | 550 | 94% | 729 | 91% |
|  | Missing | 0 |  | 2 |  |
| PR status CNB | Negative (<1%) | 57 | 18% |  |  |
|  | Positive (≥1%) | 259 | 82% |  |  |
|  | Missing | 316 |  |  |  |
| PR status PAD | Negative (<1%) | 91 | 16% | 125 | 16% |
|  | Positive (≥1%) | 495 | 84% | 673 | 84% |
|  | Missing | 0 |  | 2 |  |
| Proliferation index Ki67 (%) CNB, median (range) | | 22 (1.0–95) |  |  |  |
|  | Missing | 274 |  |  |  |
| Proliferation index Ki67 (%) PAD, median (range) | | 24 (2.5–93) |  | 15 (0–94) |  |
|  | Missing | 0 |  | 48 |  |

= preoperative data

= postoperative data

Abbreviations: CNB: core needle biopsy; ER: estrogen receptor; PAD: pathological-anatomical diagnosis; PR: progesterone receptor
