# Supplementary material for: Retrospective validation study of an artificial neural network-based preoperative decision-support tool for noninvasive lymph node staging (NILS) in women with primary breast cancer (ISRCTN14341750)
Source: BMC Cancer. 2024 Jan 16;24:86. doi: 10.1186/s12885-024-11854-1 (PMC10790472; doi:10.1186/s12885-024-11854-1)
Supplement: Supplementary file 2 — Additional file 2. Supplementary File 2 [file 12885_2024_11854_MOESM2_ESM.docx]

Definition of test characteristics for the noninvasive lymph node staging (NILS) prediction model

|  | N+ (pathology) | N0 (pathology) |
| --- | --- | --- |
| Red = the NILS model predicts nodal metastases (“test positive”) | A | B |
| Green = the NILS model predicts healthy axilla (“test negative”) | C | D |

**A** True positive (**TP**)

**B** False indication of metastatic SLN (false positive (**FP**))

**C** False indication of benign SLN (false negative (**FN**)).

This is the most hazardous situation. To provide an oncologically safe model, the cut-off for the NILS model is chosen to keep **C** low.

**D** True negative (**TN**)

The false negative rate (FNR) in the NILS model was calculated as the number of false N0 cases predicted by NILS divided by the number of cases with pathology verified axillary nodal metastasis

$$FNR=\frac{FN}{FN+TP}$$

Observed counts in the validation study:

|  | N+ (pathology) | N0 (pathology) |
| --- | --- | --- |
| Red = the NILS model predicts nodal metastases (“test positive”) | 136 | 299 |
| Green = the NILS model predicts healthy axilla (“test negative”) | 18 | 133 |
