# Supplementary material for: Retrospective validation study of an artificial neural network-based preoperative decision-support tool for noninvasive lymph node staging (NILS) in women with primary breast cancer (ISRCTN14341750)
Source: BMC Cancer. 2024 Jan 16;24:86. doi: 10.1186/s12885-024-11854-1 (PMC10790472; doi:10.1186/s12885-024-11854-1)
Supplement: Supplementary file 3 — Additional file 3. Supplementary File 3 [file 12885_2024_11854_MOESM3_ESM.docx]

Supplementary File 3. Ultrasound characteristics at diagnosis, total, and split according to study site. When there was missing data on mammography, ultrasound features were entered into the noninvasive lymph node staging (NILS) web interface.

|  | |  | | | | | |  |
| --- | --- | --- | --- | --- | --- | --- | --- | --- |
|  |  | Total | | Site 1 | | Site 2 | |  |
|  |  | Count | % | Count | % | Count | % | p-value* |
| Multifocal cancer (ultrasound) | No | 507 | 86.5% | 347 | 87.8% | 160 | 83.8% | 0.175 |
|  | Yes | 79 | 13.5% | 48 | 12.2% | 31 | 16.2% |  |
|  | Missing | 0 |  | 0 |  | 0 |  |  |
| Largest tumor (long axis, mm, ultrasound) | Mean (range) | 15.9 (3-110) | | 14.7 (3-110) | | 18.3 (4-100) | | <0.001 |
|  | Missing | 36 |  | 26 |  | 10 |  |  |
| Centrally positioned tumor (ultrasound, sub areolar or within 2 cm of the mammilla) | No | 465 | 89.6% | 310 | 91.7% | 155 | 85.6% | 0.031 |
|  | Yes | 54 | 10.4% | 28 | 8.3% | 26 | 14.4% |  |
|  | Missing | 67 |  | 57 |  | 10 |  |  |

*For categorical variables, Chi-square test was used and for continuous variables, t-test was used.
