# Supplementary material for: Retrospective validation study of an artificial neural network-based preoperative decision-support tool for noninvasive lymph node staging (NILS) in women with primary breast cancer (ISRCTN14341750)
Source: BMC Cancer. 2024 Jan 16;24:86. doi: 10.1186/s12885-024-11854-1 (PMC10790472; doi:10.1186/s12885-024-11854-1)
Supplement: Supplementary file 4 — Additional file 4. Supplementary Figure 1 [file 12885_2024_11854_MOESM4_ESM.docx]

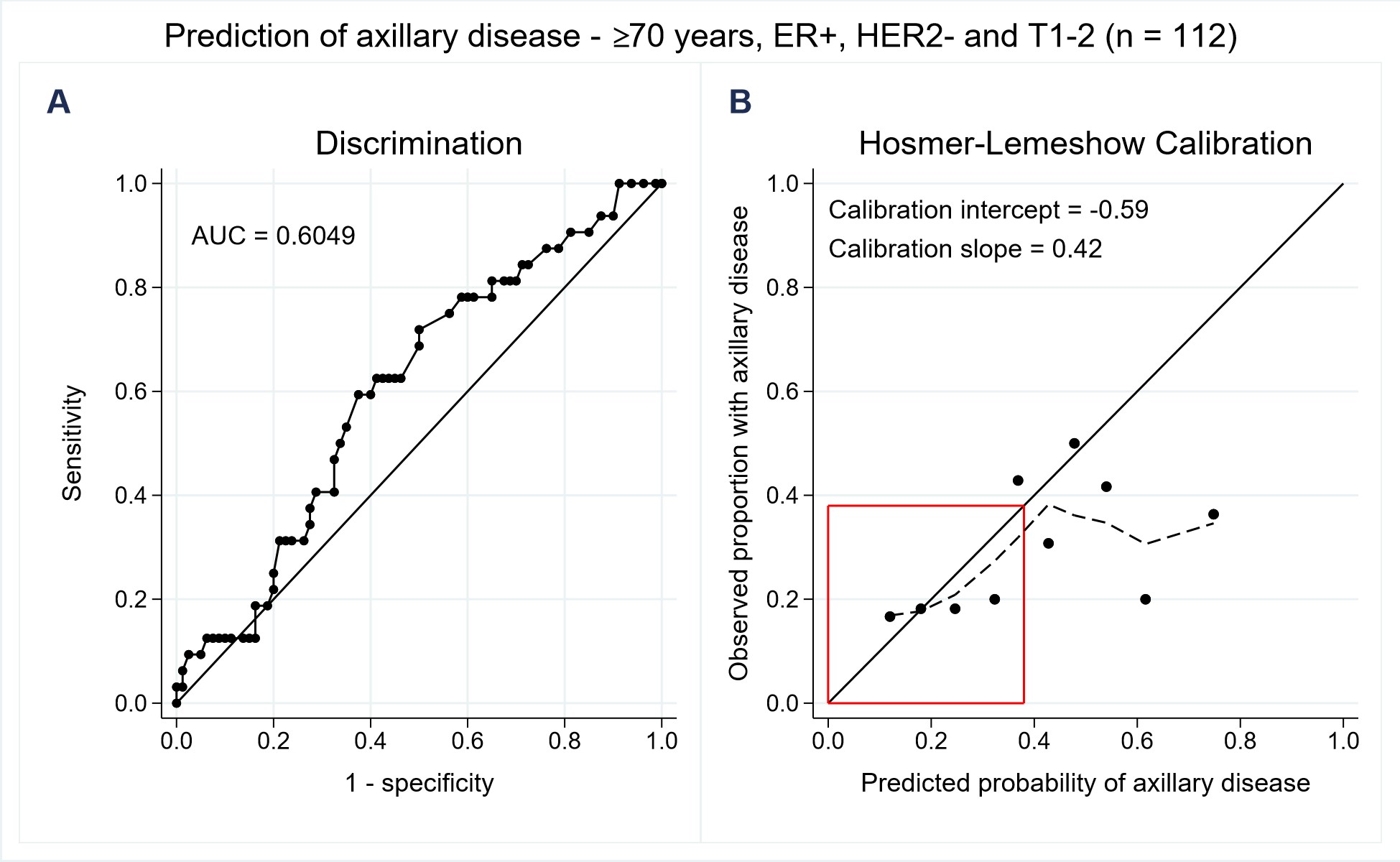


Supplementary Figure 1. Subgroup of patients aged ≥70 years with ER+, HER2-, and T1-2 tumors. A) Area under the receiver operating characteristics curve (AUC) visualizing the discriminatory performance of noninvasive lymph node staging (NILS) model for the estimation of axillary disease (N+). B) Hosmer-Lemeshow calibration plot of the observed proportion N+ versus the mean predicted probability of N+ for each decile of the predictions. Locally weighted scatterplot smoothing (LOWESS), the dotted line, was used to capture the calibration performance for low probabilities of N+, i.e. within the red box.
