# Supplementary material for: Retrospective validation study of an artificial neural network-based preoperative decision-support tool for noninvasive lymph node staging (NILS) in women with primary breast cancer (ISRCTN14341750)
Source: BMC Cancer. 2024 Jan 16;24:86. doi: 10.1186/s12885-024-11854-1 (PMC10790472; doi:10.1186/s12885-024-11854-1)
Supplement: Supplementary file 6 — Additional file 6. Supplementary Figure 3 [file 12885_2024_11854_MOESM6_ESM.docx]

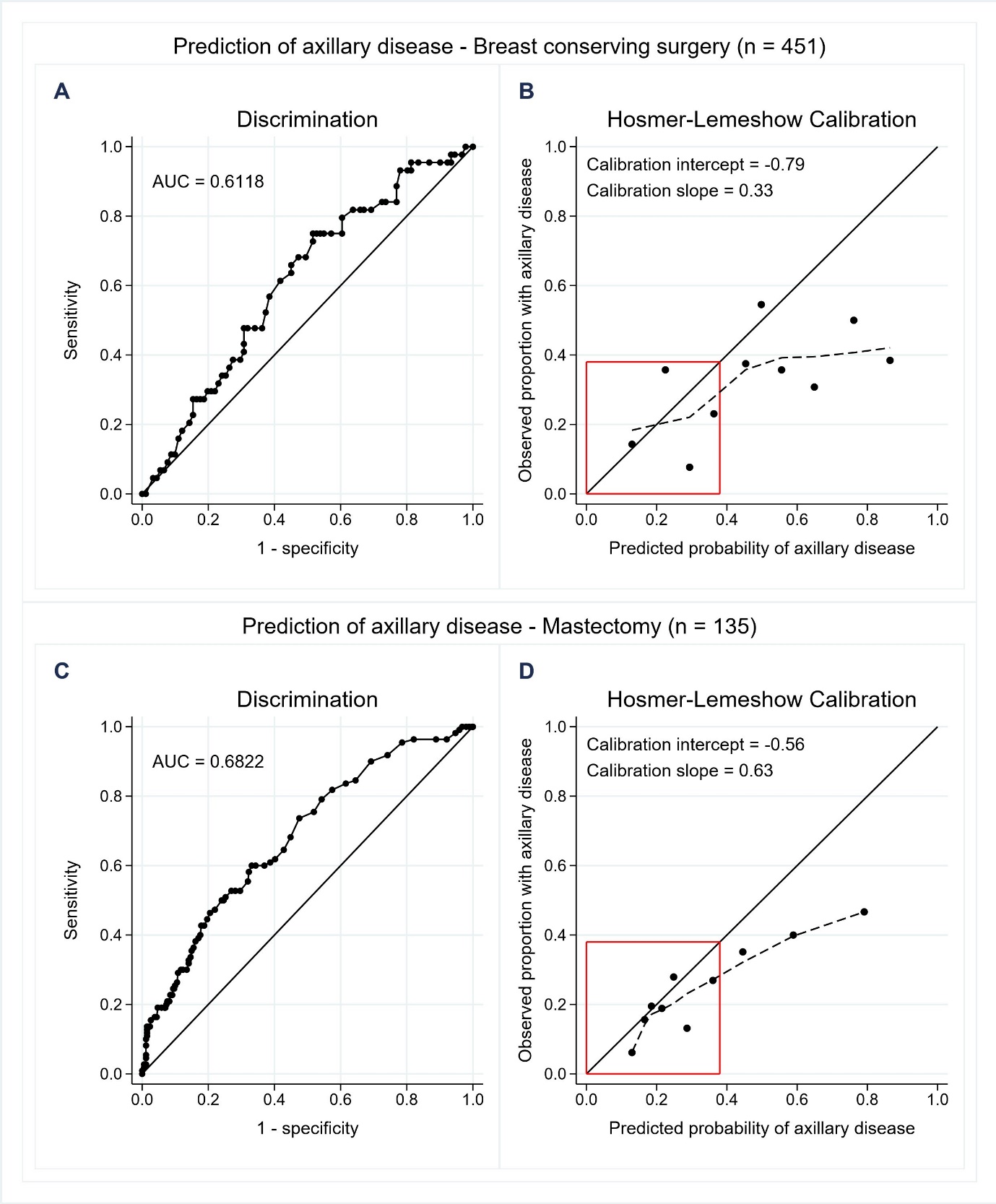


Supplementary Figure 3. Type of breast surgery (breast conserving surgery and mastectomy). A/C) Area under the receiver operating characteristics curve (AUC) visualizing the discriminatory performance of noninvasive lymph node staging (NILS) model for the estimation of axillary disease (N+). B/D) Hosmer-Lemeshow calibration plot of observed proportion N+ versus mean predicted probability of N+ for each decile of the predictions. Locally weighted scatterplot smoothing (LOWESS), the dotted line, was used to capture the calibration performance for low probabilities of N+, i.e. within the red box.
